# Systems approach for exploring the intricate associations between sweetness, color and aroma in melon fruits

**DOI:** 10.1186/s12870-015-0449-x

**Published:** 2015-03-03

**Authors:** Shiri Freilich, Shery Lev, Itay Gonda, Eli Reuveni, Vitaly Portnoy, Elad Oren, Marc Lohse, Navot Galpaz, Einat Bar, Galil Tzuri, Guy Wissotsky, Ayala Meir, Joseph Burger, Yaakov Tadmor, Arthur Schaffer, Zhangjun Fei, James Giovannoni, Efraim Lewinsohn, Nurit Katzir

**Affiliations:** Newe Ya’ar Research Center, Agricultural Research Organization, Ramat Yishay, 30095 Israel; Targenomix GmbH, Potsdam, Germany; Migal Research Institute, Kiryat Shmona, 11016 Israel; USDA-ARS and Boyce Thompson Institute for Plant Research, Cornell University, Ithaca, New York USA

**Keywords:** Fruit quality, Specialized metabolites, Metabolomic, Transcriptomic, Correlation analysis, Recombinant inbred lines

## Abstract

**Background:**

Melon (*Cucumis melo*) fruits exhibit phenotypic diversity in several key quality determinants such as taste, color and aroma. Sucrose, carotenoids and volatiles are recognized as the key compounds shaping the above corresponding traits yet the full network of biochemical events underlying their synthesis have not been comprehensively described. To delineate the cellular processes shaping fruit quality phenotypes, a population of recombinant inbred lines (RIL) was used as a source of phenotypic and genotypic variations. In parallel, ripe fruits were analyzed for both the quantified level of 77 metabolic traits directly associated with fruit quality and for RNA-seq based expression profiles generated for 27,000 unigenes. First, we explored inter-metabolite association patterns; then, we described metabolites versus gene association patterns; finally, we used the correlation-based associations for predicting uncharacterized synthesis pathways.

**Results:**

Based on metabolite versus metabolite and metabolite versus gene association patterns, we divided metabolites into two key groups: a group including ethylene and aroma determining volatiles whose accumulation patterns are correlated with the expression of genes involved in the glycolysis and TCA cycle pathways; and a group including sucrose and color determining carotenoids whose accumulation levels are correlated with the expression of genes associated with plastid formation.

**Conclusions:**

The study integrates multiple processes into a genome scale perspective of cellular activity. This lays a foundation for deciphering the role of gene markers associated with the determination of fruit quality traits.

**Electronic supplementary material:**

The online version of this article (doi:10.1186/s12870-015-0449-x) contains supplementary material, which is available to authorized users.

## Background

Fruit quality is determined by numerous traits including sweetness, color, aroma, acidity and firmness. These traits are shaped during the complex process of ripening, which although vary among species, is yet associated with typical cellular activity [1-4]. Color changes, for example, are due to alterations in chlorophyll, carotenoid and other pigment content of the plastids and vacuoles [5-7]. Sweetness in the mature fruit is the outcome of elevation in the level of mono- and disaccharides due to starch degradation or extracellular transport. Alterations in the metabolism of organic acids and generation of volatile compounds that produce aroma are common and softening is brought about by progressive degradation of cell wall components [8].

Overall, ripening changes involve a multiplicity of biochemical, metabolic, and molecular changes that have been shown to be related to alterations in the activity of specific enzymes or complete pathways. These changes lead to the accumulation of soluble sugars, organic acids, volatiles and additional specialized metabolites [9-14]. Ripening processes are not necessarily co-regulated and they are typically classified as ethylene-dependent and independent whereas the scope of ethylene control differs between species [15-17]. Here, we aim at exploring the intricate associations between prominent determinants of quality in the ripe fruit – sweetness, color and aroma. Notably, all three traits are clearly associated with the accumulation of primary or specialized metabolites, e.g., sugars, pigments, and volatiles, respectively. Hence, combining transcriptomic and metabolomic information allows the exploration of the cellular processes determining selected, quantitatively characterized, phenotypes, as well as the gene expression variations underlying the observed diversity. To date, next generation sequencing and metabolomics technologies are revolutionizing variation studies in crop by allowing the massive, simultaneous, characterization of metabolite and gene expression data from an entire, phenotypicaly diverse, populations across a range of developmental stages [18-28]. In particular, the relatively straightforward construction of Recombinant Inbred Line (RIL) populations – that is populations that are composed of the close to homozygous progeny of phenotypically diverse parents [29] – facilitates the exploration of intra-population diversity. In this project, we made use of a RIL population from melon (*Cucumis melo* L.) – a plant whose fruits generally have a sweet aromatic flavor, with great diversity in size (50 g to 15 kg), flesh color (orange, green, white, and pink), rind color (green, yellow, white, orange, red, and gray), form (round, flat, and elongated), and dimension (4 to 200 cm) [30-32]. This phenotypic diversity is associated with variability in the maturation processes where, unlike most plant species that exhibit a single ripening mode, melon fruits can be either climacteric or non-climacteric (ripening associated and not-associated with ethylene respiration bursts, respectively) [3]. Accordingly, the regulation of ripening, which in climacteric fruits is typically ethylene-dependent, in melon [7] seems to be pleiotropic and processes are classified as ethylene dependent and independent ones. Whereas the accumulation of some aroma compounds (especially esters) is thought to be ethylene dependent, the color in the mature fruit is suggested to be ethylene independent [16].

To date, the global picture of the associations between fruit quality traits and the regulatory and biochemical pathways participating in ethylene dependent and ethylene independent ripening processes have not yet been comprehensively characterized. Though sucrose, β-carotene and a selected group of volatiles were recognized as critical determinants of sweetness, color and aroma in melon, respectively [33-36], the full network of biochemical events underlying their synthesis, as well as the intricate associations between the metabolite-specific pathways, have not been comprehensively described. To delineate the cellular processes shaping fruit quality phenotypes, a population of 96 recombinant inbred lines was used as a source of defined phenotypic and genotypic variations. Ripe fruits were analyzed for the level of 77 metabolic traits directly associated with fruit quality (accumulation level of 76 metabolites and pH) and for RNA-seq based expression profiles generated for ~27,000 unigenes. First, we have explored inter-metabolite association patterns aiming to cluster together metabolites that are associated with sweetness, color or aroma and to characterize negative and positive intra-group associations of the patterns of accumulation. Then, we described metabolites versus gene association patterns aiming at associating cellular processes with selected quality traits.

## Results and discussion

### Characterizing the phenotypic diversity of key fruit quality traits in the population studied

**Figure 1:**
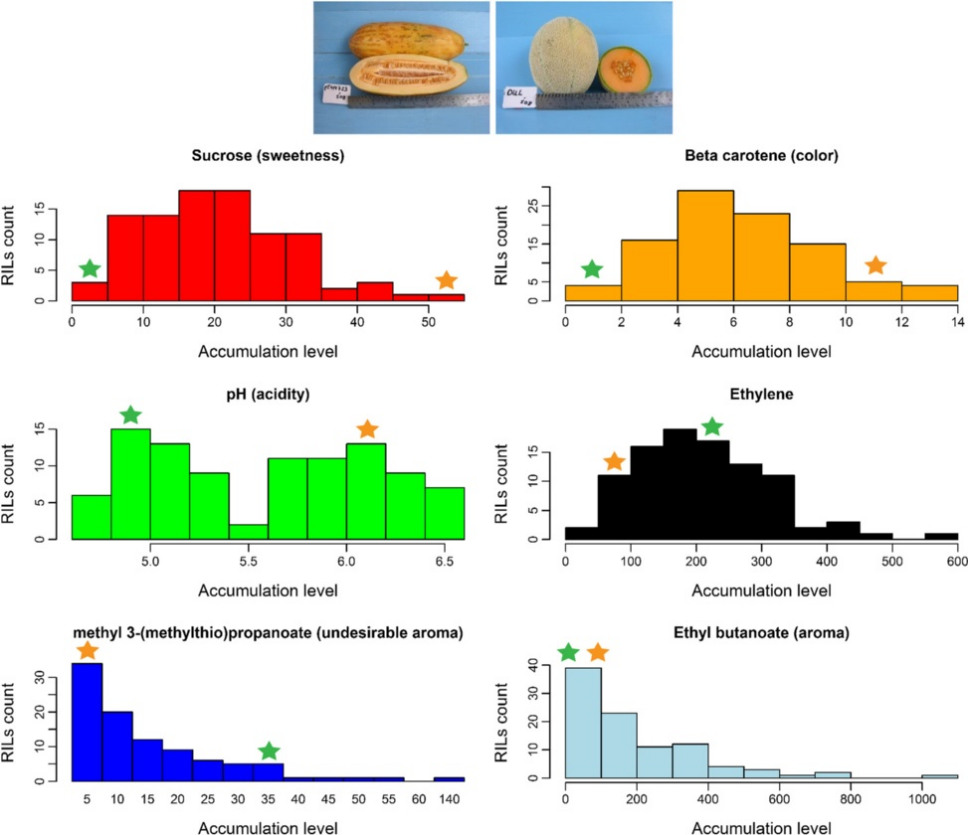
**Diversity of fruit quality associated traits within the RIL population.** Top: Ripe fruit of the parental lines PI 414723 (left) and Dulce (right). Bottom: Distribution values of selected metabolites across the RILs population. Parental values are shown at green (PI 414723) and orange (Dulce). Accumulation values across RILs are provided at Additional file 3; units are as detailed for parental values. Parental values (PI 414723/Dulce): sucrose: 4.5/52.1 mg/g; β-carotene: 1.4/9.7 ug/g F.W.; pH: 4.6/6.6; ethylene: 235/54 ppm/kg/hour; methyl 3-(methylthio)propionate: 0/34 ng compound/gr F.W.; ethyl butanoate: 4.6/23.3 ng compound/gr F.W.

Variations at the level of selected metabolites were characterized in a 96 RIL population designed to express variations in fruit quality phenotypes (Methods): fruits of the ‘Dulce’ parent are sweet and aromatic with orange flesh while those of the PI 414723 parent are non-sweet and sour and have an undesirable sulfurous aroma and very light orange flesh (Figure 1). Both lines are climacteric, yet, the PI 414723 is extremely so, characterized by fast maturation and immediate softening. To study the phenotypic diversity within their progeny population we have measured the accumulation level of metabolites that were shown to have a key role in setting the typical aroma (ethyl butanoate) [37], undesirable aroma (methyl 3-(methylthio)propionate), climacteric ripening (ethylene), sweetness (sucrose), color (β-carotene) and acidity (pH) in the melon fruit (Methods). High and low values of accumulation were recorded for ‘Dulce’ and PI 414723 according to fruit phenotypes (Figure 1). For sucrose, β-carotene, ethylene, and methyl 3-(methylthio)propionate, accumulation levels across the population are mostly within the range set by the phenotypically diverse parents (97%, 88%, 58% and 94%, respectively). For ethyl butanoate, higher values than the parental range were recorded for 94% of the RILs, possibly reflecting a heterosis vigor effect. For the pH phenotype, known to be a single-gene trait [30,38], we observed two, parent-related, peaks, unlike the distribution of the other accumulation patterns that are characteristics of polygenic quantitative traits and reflects the mosaic nature of the progeny population. The variations within this population allow exploring the intricate associations between the accumulation levels of these fruit-quality determining metabolites.

### Metabolite versus metabolites correlation patterns

**Figure 2:**
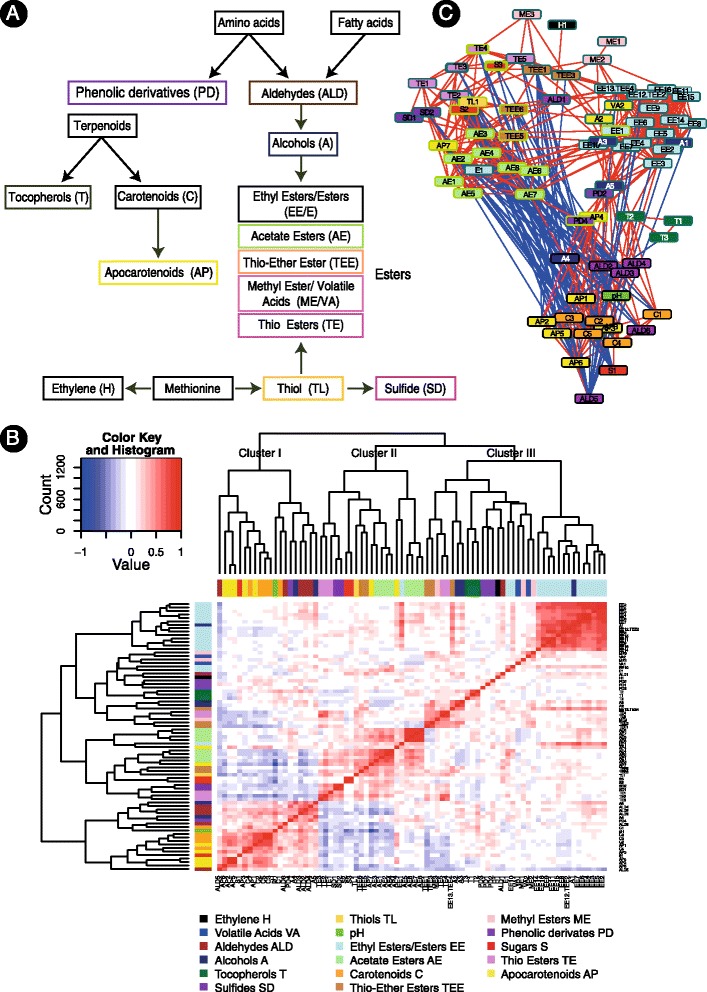
**Associations between metabolites accumulation and cellular processes.** Accumulation values were recorded for 76 metabolites directly associated with fruit quality (sweetness, color, aroma) as well as pH values (acidity). **(A)** Illustration of the proposed synthesis pathways of the metabolites in analysis. **(B)** Metabolites *versus* metabolites correlation matrix (Spearman’s rho coefficient). **(C)** Metabolites versus metabolites network. The network describes 403 positive associations (red) and 87 negative associations (blue). The layout of the network visualizes the clusters in the data (Methods). Nodes fill color is according to biochemical groups (as in panel **B**); border color is according to the clusters in panel **B** (Cluster I –black; Cluster II – light green; Cluster III – light blue). The full names of the metabolites are listed in Table 1.

To carry a comprehensive analysis of fruit-quality associated metabolites, we further defined additional 71 metabolites that are products or intermediates in the pathways involved in the production of specialized metabolites that are associated with fruit quality (Methods). The 76 metabolites and their classification into key metabolic categories are listed in Table 1; the biosynthesis associations between the categories are illustrated in Figure 2A. All metabolites show a significant genetic effect with heritability levels varying between the categories (Methods), similarly to [39].

**Table 1:** **Full name, abbreviation, and classification into metabolic category of the metabolites analyzed**

**Metabolic category**	**Metabolite identifier**	**Metabolite name**
Alcohols	A1	Ethanol
	A2	2-(methylthio)ethanol
	A3	3(methylthio)propanol
	A4	Benzyl alcohol
	A5	Benzenepropanol
Acetate Esters	AE1	Cinnamyl acetate
	AE2	Benzyl acetate
	AE3	Phenethyl acetate
	AE4	3-phyenyl-1-propanol acetate
	AE5	2-methylbutyl acetate
	AE6	2-methylpropyl acetate
	AE7	Butyl acetate
	AE8	Hexyl acetate
Aldehydes	ALD1	3-(methylthio)propanal
	ALD2	Benzylaldehyde
	ALD3	Phenylacetaldehyde
	ALD4	(*E*)-cinnamaldehyde
	ALD5	Hexanal
	ALD6	2,6-(*E*,*Z*)-nonadienal
Apocarotenoids	AP1	6-methyl-5-hepten-2-one (MHO)
	AP2	β-cyctocitral
	AP3	α-ionone
	AP4	Geranyl acetone
	AP5	β-ionone
	AP6	β-ionone epoxide
	AP7	β-(*E*)-damascenone
Carotenoids	C1	Lutein
	C2	β-carotene
	C3	ζ-carotene
	C4	α-carotene
	C5	Phytoene
Esters	E1	Benzyl propanoate
Ethyl Esters	EE1	Ethyl acetate
	EE2	Ethyl propanoate
	EE3	Ethyl 2-methyl propanoate
	EE4	Ethyl butanoate
	EE5	Ethyl 2-methylbutanoate
	EE6	Ethyl pentanoate
	EE7	Ethyl hexanoate
	EE8	Ethyl heptanoate
	EE9	Ethyl octanoate
	EE10	Ethyl benzoate
	EE11	Ethyl decanoate
Ethyl Esters/Thio-Ester Ester	EE12/TEE2	Ethyl (methylthio) acetate
	EE13/TEE4	Ethyl (3-methylthio)propanoate
Ethyl Esters	EE14	Ethyl tiglate
	EE15	Ethyl phenyl acetate
	EE16	Ethyl cinnamate
Ethylene	H1	Ethylene
Methyl Esters	ME1	Methyl benzoate
	ME2	Methyl cinnamate
	ME3	Methyl-2-methylbutanoate;
Phenolic Derivatives	PD1	Benzenacetonitrile
	PD2	Eugenol
	PD3	Phenetyl alcohol
	PD4	Cinnamyl alcohol
pH	pH	pH
Sugars	S1	Sucrose
	S2	Glucose
	S3	Fructose
Sulfide	SD1	Dimethyl disulfide
	SD2	Dimethyl trisulfide
Tocopherols	T1	δ-tocopherol
	T2	γ-tocopherol
	T3	α-tocopherol
Thio Esters	TE1	*S*-methylthio acetate
	TE2	*S*-methyl propanethioate
	TE3	*S*-methyl 2-methylpropanethioate
	TE4	*S*-methyl thiobutanoate
	TE5	*S*-methyl 2-methylbutanethioate
Thio-Ester Ester	TEE1	Methyl 2-methylthio acetate
	TEE3	Methyl 3-(methylthio)propionate
	TEE5	3-(methylthio)propyl acetate
	TEE6	2-(methylthio) ethyl acetate
Thiol	TL1	Methanethiol
Volatile Acids	VA1	Benzoic acid
	VA2	Cinnamic acid

Accumulation levels measured across the entire population were used for constructing a metabolite *versus* metabolite correlation matrix (Figure 2B). We observed a high similarity between the accumulation levels of metabolites with similar chemical structures, catalyzed by sequential or analogous catabolic reactions within the same pathway. For example, ethyl-esters form an homogenous cluster, also including ethanol, their alcoholic precursor [40]. Similarly, carotenoids are co-clustered together with their down-stream derived-volatiles, apocarotenoids [41].

Whereas intra-group associations, reflecting biochemical structure and common bio-synthesis pathways, can be expected, we further explored the inter-groups association patterns aiming to gain a more global view on ripening processes in melon. According to the patterns of distribution we divided metabolites into 3 key clusters (Methods), grouping together metabolites with similar levels of accumulation across RIL (Additional file 1). Sucrose clusters together with carotenoids, apocarotenoids and aldehydes, also associated with a typical high pH (Cluster I); glucose and fructose cluster together with acetate esters and some thioesters (Cluster II); ethyl-esters cluster together with their alcoholic precursors and ethylene (Cluster III). Complementary to the hierarchical clustering analysis, we also visualized significant metabolite-to-metabolite associations (correlation coefficient rho > = |0.3|, Methods) via a network of nodes (metabolites) and edges (either positive correlations in red or negative correlations in blue), taking a similar approach to [28]. The layout of the network, whose topology shows the clustered-structure of a graph (Methods), illustrates the stratification of metabolites into biochemical groups (Figure 2C). In accordance with the pattern observed in the hierarchical clustering, 83% of the significant positive associations and none of the negative associations occur within the clusters; more significant positive associations are formed between metabolites from Clusters II and III, in comparison to the number of positive associations formed with metabolite members of Cluster I (Additional file 1).

Considering the traits associated with the different groups, metabolites in Cluster I are mainly associated with determination of sweetness, color and acidity. An association between sucrose and carotenoid accumulation was previously demonstrated when sucrose deficiency lead to inhibition of carotenoid accumulation in fruits [42]. Metabolites in Clusters II and III are associated with assessing desirable, melon-typical aroma (for example the ethyl-esters ethyl butanoate, ethyl 2-methylpropanoate, methyl-2-methylbutanoate and ethyl 2-methylbutanoate) [43], and undesirable aroma (for example methanethiol, sulfides and thioesters), respectively. The classification of fructose and glucose into Cluster II corresponds with previous studies describing an inverse association between their level of accumulation versus the accumulation level of sucrose (Cluster I) during fruit ripening [44-46].

Overall, some of the associations detected are likely to reflect the synthesis pathways while others are possibly the outcome of genomic association or common regulatory processes. For example, aldehydes, primary precursors in the synthesis of volatile alcohols and esters (Figure 2A), are found in Cluster I, where the more downstream volatiles (alcohols and esters, Figure 2A) are co-located in Clusters II and III, where 30 negative associations are detected between these compound groups (Figure 2C). The co-classification of esters together with alcohols corresponds with the documented association between the total amounts of esters and alcohols in ripe fruits [44]. The co-classification of ethylene with the volatile esters possibly reflects its demonstrated role in controlling their production [13] by regulating the reduction of aldehydes into alcohols which in turn are converted into esters [16]. Accordingly, aldehydes – whose production is not directly controlled by ethylene - are found in the ethylene non associated cluster (Cluster I), together with most of the non-volatile compounds. Similarly, the lack of similarity in the accumulation patterns of ethylene *versus* sugars and carotenoids (Cluster I members) suggests that flesh pigmentation (as the outcome of carotenoid accumulation) and sweetness level in the melon fruit are not directly controlled by ethylene. Hence, the clustering pattern observed provides a corroborative support to the model suggested by Ayub *et al*. [7], dividing fruit ripening processes in melon to ethylene dependent and independent ones. Whereas the accumulation of the aroma compounds volatile esters (Cluster II and III) is thought to be ethylene dependent, the color in the mature fruit (Cluster I) appears to be ethylene independent.

### Using accumulation and expression patterns for linking metabolites with genes

Making use of the gene expression data, extracted in parallel to the metabolite accumulation data, we have calculated the correlations between all gene-metabolite combinations (Methods). In order to validate the correspondence between the computed expression-accumulation associations and previously reported empirical observations, we focused on the group of volatile esters, a class of compounds contributing to the aroma of melon fruit [43,44,47]. The production of esters is catalyzed by alcohol acyl-transferases (Cm-AATs) through the esterification of an alcohol and acyl-CoA substrates [10]. Different Cm-AAT enzymes use different substrates (alcohol and/or acyl-CoA) to produce different ester products [11,13]. Product and substrate specificities for some of these genes have been described in detail in *in vitro* systems for a set of volatile esters, ten of them included in our data set [13]. Eight of these ten esters were shown to be produced, at varying amounts, by *Cm-AAT1* (enzyme product of MELO3C024771) and none of the esters were produced by *Cm-AAT2* (enzyme product of MELO3C024766). In order to examine whether metabolite-gene correlation analyses are indicative of potential biochemical association, we looked at the correlations between the accumulation levels of the ten volatile esters and the expression levels of *Cm-AAT1 and 2* (Table 2). For *Cm-AAT1* we observe a significant positive correlation (*p* value < 0 .05 in a Spearman rank correlation) with five esters, all produced by Cm-AAT1 [13]; most of the remaining (4 esters, Table 1), non correlated esters, were shown either not to be produced by Cm-AAT1 or to be produced at low levels (<100 mg^−1^). A homolog of Cm-AAT2 that in previous studies was regarded as catalytically inactive [13] is not correlated with any of these metabolites. Overall, in all 3 cases where a high level of volatile ester was experimentally detected (>1000 mg^−1^, Table 2), we also observe a significant gene expression-product accumulation correlation; in all 12 cases where a production of volatile esters was not experimentally detected (either by Cm-AAT1 or Cm-AAT2), a significant correlation was not observed; also, a significant correlation was not observed in two out of three cases of low production. This overall agreement between the predictions and laboratory tests encourages the use of the gene-correlation associations in order to predict unknown synthetic pathways. For example, looking for the metabolite associations of the *CmAAT2* coding sequence we found six metabolites that are significantly positively correlated with CmAAT2 expression including ethyl esters, thio esters, and thio ethyl esters. Though correlation cannot be regarded as a conclusive indication for a synthetic role, these associations provide a testable set of predictions for potential function of the CmAAT2 whose biochemical role has not been yet elucidated.

**Table 2:** **Gene-metabolite correlation values between alcohol acyl-transferases versus experimental evidence for their metabolic association**

**Ester product**	ID accession*	***Cm-AAT1*** **(MELO3C024771)**		***Cm-AAT2*** **(MELO3C024766)**	
		**Rho correlation coefficient (** ***p*** **value)ζ**	**Production levels in vitro§**	**Rho correlation coefficient (** ***p*** **value)ζ**	**Production levels in vitro§**
Butyl acetate	AE7	0.34 (0.0009)	High	NS	ND
Hexyl acetate	AE8	0.29 (0.004)	High	NS	ND
2-methylpropyl acetate acetate	AE6	0.34 (0.0008)	Low	NS	ND
Benzyl acetate	AE2	0.28 (0.006)	High	NS	ND
Cinnamyl acetate	AE1	0.41 (3.43e-05)	Medium	NS	ND
Ethyl phenyl acetate	EE15	NS	Medium	NS	ND
Ethyl propanoate	EE2	NS	ND	NS	ND
Ethyl butanoate	EE4	NS	Low	NS	ND
Ethyl 2-methyl propanoate	EE3	NS	ND	NS	ND
Ethyl hexanoate	EE7	NS	Low	NS	ND
Ethyl heptanoate	EE8	NS	NA	0.22 (0.03)	NA
Ethyl octanoate	EE9	NG	NA	0.22 (0.03)	NA
Ethyl (methylthio) acetate	EE12/TEE2	NS	NA	0.24 (0.02)	NA
*S*-methyl 2-methylpropanethioate	TE3	NS	NA	0.20 (0.04)	NA
Methyl 2-methylthio acetate	TEE1	NG	NA	0.26 (0.008)	NA
Methyl 3-(methylthio)propionate	TEE3	NG	NA	0.21 (0.008)	NA

Upper part of the table: Metabolic evidence is based on *in vitro* experiments [13] testing the product specificity of Cm-AAT1-4. The ten metabolites in the upper part of the table were retrieved from the crossing of the 29 ester products tested at [13] with our set of metabolites. *Cm-AAT3* (MELO3C024769 and MELO3C024762) and *Cm-AAT4* (MELO3C017688) were detected at very low levels at the ripe fruit across the RIL population (Additional file 4) hence correlation values were not computed Median expression values (RPKM) across the RIL population for MELO3C024771 (*Cm-AAT1*), MELO3C024766 (*Cm-AAT2*), MELO3C024769 (*Cm-AAT3*), MELO3C024762 (*Cm-AAT3*) and MELO3C017688 (*Cm-AAT4*), respectively: 6777.400, 2881.290, 0.075, 0.840 and 0.000. Lower part of the table: metabolites (out of the 76 metabolites in the data set) with significant (<0.05) positive correlation with *CmAAT2*. The catalytic ability of CmAAT2 to produce the 6 metabolites was not tested at [13]. In vitro assays not carry at the reference work [13] are marked as NA.

*Full names are as in Table 1.

ζNS – not significant (*p* value > 0.05); NG – negative correlation; NA - In vitro assays not carry at the reference work [13].

§According to [13]. High production levels: > 1000 mg^−1^; medium production levels: 100–1000 mg^−1^; low production levels: < 100 mg^−1^; ND – not detected.

### *Functional analysis of metabolite-gene* associations

Beyond the identification of specific, uncharacterized, pathways, we further aimed at the comprehensive characterization of the associations between genes and metabolites. Though, for some compound groups such as sucrose and carotenoids, we did not expect to observe a correlation between their pattern of accumulation and the expression level of genes directly involved in their synthesis [36,48], we did aim at delineating the overall cellular activity that is typical of their enhanced, or slowed-down, production. To this end, we recorded for each metabolite its list of associated genes (Methods). The genes were assigned to the MapMan hierarchical annotation scheme (Methods) providing high and low levels description for their functional role. MELO3C010686, for example, is assigned to the “amino acid metabolism” category at the highest classification level, “synthesis” at the second level, “central amino acid metabolism” at the third level, “alanine” at the fourth level, and “alanine aminotransferase” at the fifth level. For each metabolite, we calculated the frequency of genes at each classification level in order to outline these cellular processes that are more significantly associated with its rate of production (Methods).

**Figure 3:**
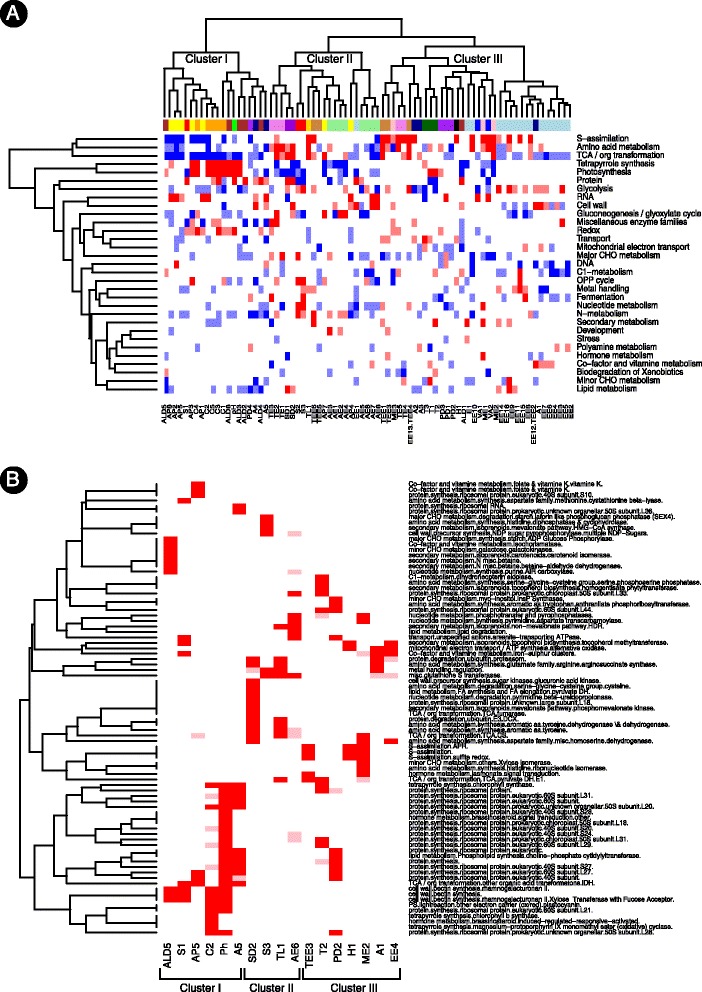
**A heatmap of over-represented categories from the metabolites versus genes correlation data.** The level of representation of genes positively and negatively correlated with metabolites across all level of MapMan categories was subjected to cumulative hypergeometric distribution tests. Categories that are significantly enriched in positively correlated genes are colored in red (light red: 0.01 < *P* value < = 0.05; dark red: < *P* value < = 0.01); Categories that are significantly enriched in negatively correlated genes are colored in blue (light blue: 0.01 < *P* value < = 0.05; dark blue: < *P* value < = 0.01). For each category (rows) numbers in bracts are indicative of its level of classification (left) and the number of genes assigned to the category (left). **(A)** Categories at the top level of classification. Metabolites are ordered according to their classification pattern at the metabolites versus metabolites analysis (Figure 2B). Representative metabolites are marked in green. **(B)** Top over-represented categories across all MapMan classification levels. Five top categories were selected for each representative metabolite according to *P* values (Methods). Only over-representation of positively-correlated genes was considered. Metabolites are ordered consequently, according to their classification pattern at the metabolites versus metabolites analysis (Figure 2B). The full names of the metabolites are listed in Table 1. In order to simplify the visualization the original 97 categories were reduced into 83 by choosing single category to represent several similar categories (considering their higher level path) sharing the same enrichment profile across metabolites. E.g., a single category was chosen from the following categories at level 7: protein.synthesis.ribosomal-protein.prokaryotic.chloroplast.50S.subunit.L28; protein.synthesis.ribosomal-protein.prokaryotic.chloroplast.50S.subunit.L10; protein.synthesis.ribosomal-protein.prokaryotic.chloroplast.30S subunit.PSRP3; and protein.synthesis.ribosomal-protein.prokaryotic.chloroplast.50S subunit.L18 – all categories showing over-representation of genes positively correlated with pH.

Taking a top-down approach, we first looked at the categories at the highest level of classification (most general). Figure 3A lists for each metabolite these categories that are significantly enriched in genes with which it is either positively or negatively correlated (red and blue coloring, respectively). Metabolites are ordered as in Figure 2 according to their co-clustering pattern, pointing at the key functional differences between the three groups. For metabolites from Cluster I, pathways associated with photo-protection activity including redox, photosynthesis, and tetrapyrrole (a precursor of chlorophyll) synthesis categories are in many cases enriched in positively correlated genes. The enrichment of carotenoid-correlated genes in these photosynthesis-related pathways was previously reported, suggested to be explained by the well documented role of carotenoids in light harvesting and photoprotection [22]. Genes from the tetrapyrrole synthesis pathway that are positively correlated with carotenoid levels include chlorophyllase (MELO3C014286) – a chlorophyll degrading enzyme, possibly accounting for the demonstrated degradation of chlorophyll accompanying the accumulation of carotenoids during ripening [6,33,41,49].

In Clusters II and III, including ethylene and many volatile compounds, pathways enriched in positively correlated genes include the TCA and glycolysis pathways. The positive enrichment in genes from the TCA and glyscolysis pathways is in accordance with the ethylene-dependent large respiratory increase during ripening, accompanied by radical alteration in the concentrations of organic acids in the TCA cycle [50]. The positive enrichment in genes from the sulfur associated pathway (S-assimilation) corresponds with the incorporation of sulfur residues in thio-ester compounds. Overall, a reverse pattern of enrichment (blue versus red) is observed between the metabolites from Cluster I versus the metabolites from Clusters II and III. Mainly, pathways enriched in genes that are negatively correlated with metabolites from Cluster I include the TCA, sulfur-assimilation and glycolysis pathways (Figure 3A).

### Delineation of key cellular processes involved in metabolite accumulation

To narrow down the big picture and identify the specific pathways associated with metabolite accumulation we focused on a subset of 17 representative metabolic traits. Metabolites (marked at green, Figure 3A) were selected to represent the main biochemical groups studied, considering both their importance for determining fruit quality traits and their clustering pattern. In cases where clustering pattern does not reflect the biochemical association, more than a single metabolite was selected. For example, since sugars fall into two clusters, both sucrose (S1, Cluster I) and fructose (S3, Cluster II) were selected as representative metabolites.

For the representative metabolites, we screened across all classification levels, looking for pathways that are enriched with positively correlated genes (Methods). Overall, we recorded 645 categories associated with at least a single metabolite (Additional file 2). To further narrow down the analysis, for each metabolite we then recorded its top five most significant categories yielding a table with 97 pathway entries (Figure 3B). The categories include a single entry at the top hierarchical level (S assimilation) and entries up to the seventh level of classification – mainly for synthesis pathways of the prokaryote ribosomal subunits of cellular organelles. Synthesis of prokaryote ribosomal subunits of cellular organelles, including the chloroplast subunits, is mainly detected for the metabolite members of Cluster I – β-carotene (C2), benzenepropanol (A5) and pH. The enhanced production of these plastid ribosomal subunits together with the elevated tetrapyrrole synthesis (Figure 3B) is possibly indicative of the increased production of chromoplasts – plastids highly similar to chloroplasts in which carotenoids are synthesized and stored.

As already detected at the highest classification level (Figure 3A), metabolites from Clusters II and III exhibit an overall similarity in the cellular activity accompanying their accumulation, where pathways detected include such belonging to TCA and S assimilation activity. At a lower level of classification, categories selected include the synthesis of aromatic amino acids, detected for the phenyl propanoid derivate eugenol (PD2, Cluster III). The increase in activity of enzymes associated with aromatic amino acids metabolism corresponds with their role as precursors of many volatiles in the melon fruit [35]. Significant degradation activity of the sulfur containing amino acids cysteine is predicted for the sulfide representative dimethyl trisulfide (SD2). This corresponds with cysteine being the central precursor of all organic molecules containing reduced sulfur ranging from the amino acid methionine to peptides as glutathione [51]. Glutathione S-transferase activity is detected among the top most significant categories for 5 metabolites in Clusters II and III (Figure 3B) and significant activity in methionine degradation is observed for four metabolites of these clusters (AE6, ME2, TEE3, TL1, Additional file 2).

### Identification of key gene-groups associated with ethylene dependent and independent processes

**Figure 4:**
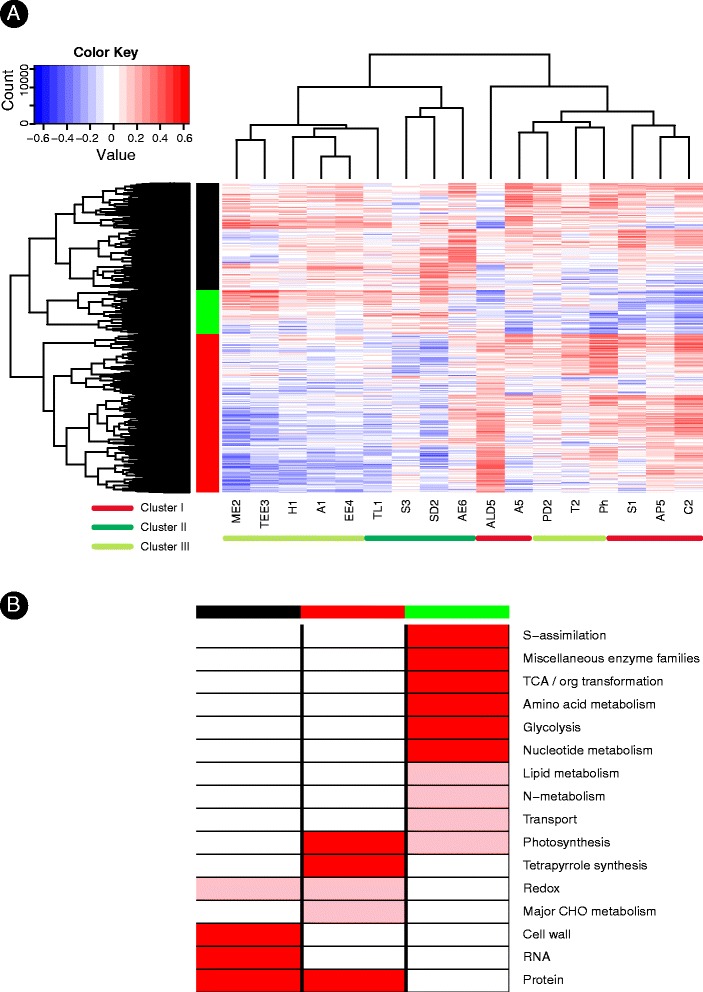
**Associating groups of genes and cellular processes accompanying the accumulation of metabolites.** The full names of the metabolites are listed in Table 1. **(A)** Metabolites versus genes correlation matrix (Spearman’s rho coefficient). The heatmap display correlation values for the 17 representative metabolic traits detailed at Figure 3B. 6129 genes were significantly correlated with at least a single representative metabolic trait (|*r*| > 0.3). The rows in the heat map are the genes clustered by their expression patterns and the columns are the metabolic traits. Gene groups (marked at green, black and red) were selected by cutting the tree. **(B)** A heat map of over-represented categories from the black, green and red gene groups marked at **(A)**. The level of representation of genes within each group across the top level of MapMan categories was subjected to cumulative hypergeometric distribution tests. Categories that are significantly enriched are colored in red (light red: 0.01 < *P* value < = 0.05; dark red: < *P* value < = 0.01). For each category (rows) numbers in bracts are indicative of its level of classification (left) and the number of genes assigned to the category (left).

Looking directly at the correlation matrix of metabolite accumulation versus gene expression, for the large majority of metabolites, the clustering pattern remains constant between the metabolite versus metabolites and metabolites versus gene matrices (Figure 4A). The co-classification of γ-tocopherol (T2, Cluster III) together with metabolites from Cluster I including carotenoids and apocarotenoids (Cluster I), can possibly reflect their common biochemical origin being all synthesized through the deoxyxylulose phosphate plastidial terpenoid pathway [40] from an isoprenoid precursor (Figure 2A). Correspondingly, cellular activities associated with the level of γ-tocopherol accumulation include categories typical to Cluster I metabolites such as the synthesis of chloroplast ribosomal proteins and tetrapyrroles (Figure 3A). The classification of tocopherols in the metabolite Cluster III (metabolite versus metabolites accumulation pattern, Figure 2B), together with ethylene, can be explained by recent evidence for the role of tocopherols in regulating ethylene signaling pathways [52]. The full correlation matrix of 77 metabolites versus gene expression is presented at Additional file 3, also showing an overall agreement between the clustering patterns of the main metabolite groups as derived from the metabolite-versus-metabolite analysis.

**Figure 5:**
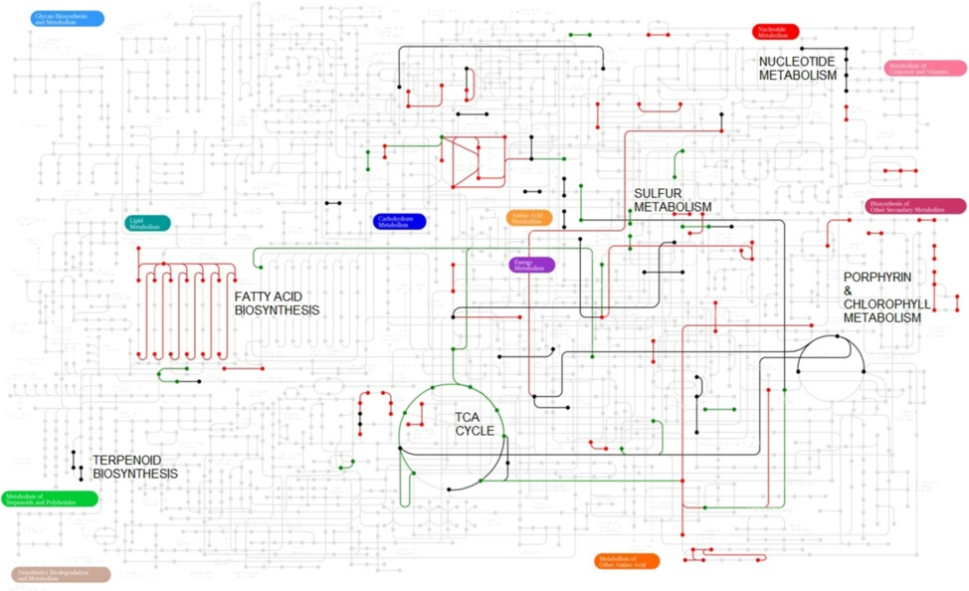
**Highlighting enzymes from three gene groups on top of the generic KEGG metabolic pathway.** Genes were classified into three groups (black, red and green) according to their clustering pattern at the metabolites versus genes correlation matrix (Figure 4). Following mapping gene accessions to ECs (Methods), only ECs that were classified into a single gene group were further highlighted.

Taking a gene rather than a metabolite perspective, the clustering pattern of the genes correlated with the representative metabolites reveals 3 key gene groups marked at black, green and red (Figure 4A). Genes from the black group show an overall correlation with most metabolites and are found to be associated with house-keeping and maintenance functions (Figure 4B); genes from the green group show reverse pattern of association versus genes from the red group where green genes are mostly associated with volatiles, sulfide derived metabolites and ethylene (Cluster II and III) and red genes are mostly associated with sweetness, color and acidity determining representatives (Cluster I). The gene-centered enrichment analysis (Figure 4B) reinforces the key observations derived at the single compound level where ethylene-associated compounds are correlated with genes involved in key energy producing pathways (glycolysis, TCA) and sulfur assimilation and ethylene non-associated compounds are correlated with plastid-related activities such as photoprotection activities. Finally, to gain a network perspective of the functional significance of the gene clusters we have highlighted enzymes from all groups on top of the generic KEGG metabolic pathway (Methods, Figure 5). Volatile-associated enzymes (green) are most dominant at the TCA, and sulfur metabolism pathways. Red enzymes, on the other hand, are involved in processes of specialized metabolism activities including chlorophyll metabolism.

## Conclusions

Here we describe an integrated transcriptomic and metabolomic data from the mature fruit of a phenotypically diverse melon population. Extensive metabolic phenotyping were previously carried in melon [27,46,47,53,54], and in other fruits [26,28], though without the parallel analysis of the transcriptomic data. Moreover, here we focused on specialized-metabolism pathways rather than primary metabolism and elemental profiling aiming to preserve a relatively direct association between metabolite and a phenotypic trait. An integrative metabolomic-transcriptomic approach was successfully applied for identifying genes that control carotenoid accumulation in the mature tomato fruit [22]. Here, we have extended the approach to additional metabolites, in order to delineate a comprehensive description of the cellular processes associated with the determination of fruit quality traits. Notably, expression measures are based on a single fruit-pool from each line (Methods), allowing a better coverage of each sample. Since we focus on gene-metabolite associations across the entire population, values rely on multiple estimates and reduce the likelihood of biases caused by technical errors.

The analysis points at two independent processes associated with melon fruit maturation: ethylene associated activity, where the synthesis of ethylene and key volatiles (Clusters II and III) is characterized by enhanced activity of enzymes involved in the TCA cycle and sulfur metabolism, including the enhanced degradation of sulfur-containing amino acids and the increased synthesis of aromatic amino acids, providing precursors to many volatiles. Ethylene non-associated processes include the accumulation of sucrose and β-carotene (Cluster I). The cellular processes accompanying their accumulation are associated with plastids formation and activity, rather than with the expression of genes directing the synthesis of these metabolites suggesting that color accumulation during ripening is to a large extent regulated at the level of plastid differentiation.

Overall, the present approach of integrated transcriptome and metabolite profiling using RILs as a source of variation of gene expression and metabolite accumulation sums together multiple past efforts to study ripening processes taking a single gene perspective. Our findings are in accordance with such previous works. Yet, taking a genome scale perspective, our study provides a comprehensive model describing the cellular activity during ripening. Moreover it suggests a testable set of solutions for the possible catalytic activity of enzymes. Considering the vast diversity of specialized metabolites in plants and the large numbers of expressed genes whose functional role is yet unknown [55,56], the demonstrated correlation between the expression level of alcohol acyl-transferases and their catalytic products, supports the use of correlation analyses for predicting substrate and product specificities of enzymes.

Notably, the role of ripening process in shaping traits associated with fruit-quality is of obvious importance. Yet, our understanding is complicated by the polygenic nature of most of these traits [36,57]. Notably, the RNA-seq data provides not only expression information but also allow the characterization of the genetic variations associated with the phenotypic diversity. A parallel use of the data for gene association analysis can provide predictions for genetic markers of selected traits. Taken together, the gene association approach and the expression based correlation analysis are complementary as they allow functional contextualization of the selected markers. For example, in cases where several markers were found to be associated with a given traits, highlighting the pathway connectivity between these markers can explain their concentrated activity as synergistic or overlapping. Hence, this analysis lays foundation for deciphering the role of gene markers associated with the determination of polygenic traits. Though the synthesis of fruit quality associated metabolites such as sugars, carotenoids and volatiles is long being explored, such system level analyses will enhance our understanding of the factors that determines their level of accumulation, and quantify the unique contribution of relevant genes, also considering the mutual impact of multiple genes all individually contributing to a given phenotype.

## Methods

### Plant material

A population of recombinant inbred lines was constructed from a cross between PI 414723-S5 (*C. melo* var *momordica*) and ‘Dulce’ cv. (*C. melo* var *reticulatus*), as described in [30,58]. Ninety six lines were grown in a complete randomized design, in an open field at Ne’we Yaar Research Center in the spring-summer season of 2010. The experimental design for cultivation and harvest was directed by standard cultivation practice [30,46,58]. Briefly, the soil type was fine clay, density of plants was 10,000 plants/ha, irrigation in the field was about 250 m^3^ per ha once a week corresponding to 25 l per plant per week, and fertilizer management and pathogen pest control were performed according to the standard practices at Newe Ya’ar. Each line was represented by 12 plants. Flowers were pollinated and tagged at anthesis and one fruit was allowed to develop per plant. The fruits were sampled at ripening, once the abscission layer was fully developed and the fruit detached when touched. Typically ripening took place 35–40 days after anthesis (DAA), in accordance with a previous study under similar conditions, in which the PI414723 fruits reached ripening in 30 DAA and ‘Dulce’ fruits in 40 DAA [24]. Flesh tissues (mesocarp) were taken from five fruits (per line) for transcriptomic and metabolomic analyses. Tissues were immediately frozen in liquid nitrogen and stored at −80°C.

### Metabolic phenotyping

77 metabolic traits (76 metabolite levels and pH) were measured across the population of RILs. For the analysis of the 64 volatiles, frozen fruit samples (3 g) were placed in a 10 ml glass vial containing 1 g solid NaCl. To each vial 5 ml of a 20% (w/v) NaCl solution and 0.3 μg of 2-heptanone, which was used as an internal standard, were added. The vial was then sealed and stored at 4°C, for no longer than 1 week until analyzed. Solid-Phase Micro-Extraction (SPME) sampling was conducted according to [59] with slight modifications. The sample was preheated to 50°C, agitated for 5 min at 500 RPM and then a 65 μm fused silica fiber coated with polydimethylsiloxane/divinylbenzene/carboxen (PDMS/DVB/CAR) SPME fiber (Supelco Inc., http://www.sigmaaldrich.com/analytical-chromatography.html) was inserted into the vial and exposed to the sample headspace. After 25 min the SPME fiber was introduced into the injector port of the GC-MS. Volatile compounds were analyzed and quantified on a GC-MS apparatus (Agilent Technologies, http://www.home.agilent.com/agilent/home.jspx?cc=US&lc=eng) according to [59]. Carotenoids and tocopherols were extracted from 0.5 g FW of finely grinded frozen fruit flesh tissue in hexane:acetone:ethanol (50:25:25, v/v/v) mixture as described in [60] and were analyzed, identified and quantified with a Waters (Milford, MA) 2695 HPLC apparatus equipped with a Waters 996 PDA detector (carotenoids) and FD detector (tocopherols), as described previously [61]. Soluble sugars were sampled using HPLC applying the procedure outlined at [62]; ethylene was sampled using GC-flame ionization detector (FID); pH of the flesh samples were measured by pH meter (PH-03(II), ZD Instrument Corp., China) in juice squeezed from the fruit. In each RIL, volatile accumulation levels were sampled in three replicates, from each fruit separately; other metabolic traits were sampled separately in five replicates. One-way ANOVA analyses were carried out for each metabolite with the RIL (genetic factor) as the main effect using REML method. Metabolites with severly skewed distribution were subject to a square-root transformation. In all 77 traits we detected a significant genetic effect (p ≤ 0.05, Additional file 4). Broad-sense heritability (H^2^) was calculated based on ANOVA component values using intra-class correlations [63] (Additional file 4). Statistic calculations were conducted in JMP® Pro, Version 10.0.2. SAS Institute Inc., Cary, NC, 1989–2014. The mean value over all fruits sampled was set as the level of accumultion in RIL. Additional file 5 provides the full list of metabolites and their accumulation levels across all lines sampled.

### Preparation of cDNA libraries and transcriptomic profiling

For each line, total RNA was extracted from pooled flesh tissues of five ripe fruits and used for the preparation of Illumina cDNA libraries [24]. RNA quality and quantity were determined using a ND-1000 spectrophotometer (Nanodrop Technologies Inc., Wilmington, DE. USA) and denaturing agarose gel electrophoresis. Strand-specific RNA-seq libraries were constructed using the protocol of [64]. Libraries were barcoded and multiplexed in collections of four samples per lane of sequencing. The libraries were barcoded and 8 to 20 libraries were pooled and sequenced on one lane of the Illumina GAII and HiSeq 2000 systems, respectively. Five to 10 million reads were obtained for each library. RNA-Seq reads were first aligned to ribosomal RNA sequence database [65] using Bowtie allowing up to two mismatches [66], to remove any possible rRNA contaminations. The resulting cleaned reads were aligned to the melon genome [67] using TopHat [68] allowing a single segment mismatch. Following alignments, raw counts for each melon gene were normalized to reads per kilobase of exon model per million mapped reads (RPKM) [69]. RPKM values for 27,427 gene accessions across the RILs population are available at Additional file 6.

### Correlation analysis of genes and metabolites and network visualization

Metabolites *versus* metabolites correlations between the 77 metabolic traits and metabolites versus genes correlations between the 27,427 gene accessions and the 77 metabolic traits were calculated using the Spearman rank correlation. RPKM floor was set to 3. A total of 9721 genes are considered to be associated with at least a single metabolite, as defined by a correlation coefficient rho > |0.3|. The heat maps of metabolites versus metabolites and metabolites versus genes were generated by two-way hierarchical clustering. Metabolites were divided into three groups using the cutree function (k = 3); genes were divided into four groups using the cutree function (k = 4).

Metabolite versus metabolites network was constructed setting a threshold of correlation coefficient rho > = |0.3| (*p* value < 0.003), similar to [28]. All computations and preparation for the network visualizations were generated in R. The graphical presentation of the network was composed with Cytoscape version 3.1.1. Network layout was carried automatically using the ‘organic’ layout option that visualizes the clusters in the data. Only positive associations were considered for the layout, and negative associations are illustrated over the formed graph.

### Gene annotations and functional analysis of gene groups

The map of functional categories was retrieved using the Mercator mapping tool of MapMan [70,71]. Overall, 13,579 genes were assigned to a bin category. Genes co-selected by transcript–metabolite correlation analysis were grouped together and the functional significance of such groups was estimated by calculating the cumulative hypergeometric *p* value for the probability that a gene group is enriched with a functional bin at a rate higher than chance expectation. Multiple tests were carried for all gene groups – functional categories at all hierarchical levels. Significance cut-off was determined by setting a False Discovery Rate threshold of 10%. The significance of the appearance rate of each functional category within each gene group is provided at Additional file 2. The significance of the appearance rate of the top functional categories at the three key gene groups (Figure 4) was determined using the same procedure. Functional categories were then converted into EC numbers using the file the file “BIN2EC” at the mapping repository http://mapman.gabipd.org/web/guest/mapmanstore. ECs were mapped to KEGG network using the KEGG Mapper.

### Availability of supporting data

Illumina sequencing data were deposited in the NCBI SRA repository under accession identifier SRP052934. All additional data files and a file describing the accumulation levels of metabolites across RILs and across biological repeats are available in the LabArchives repository [72].

## Additional files

Additional file 1:
**Provides a comparison of the intra- and inter-cluster similarities of metabolite accumulation patterns in the three clusters of metabolites, determined according to hierarchical clustering.**
Additional file 2:
**Is a table listing the significance of the occurrence rate of functional categories within gene groups selected according to their correlation with a metabolite (rho > 0.3).** Values represent the p value in a cumulative hypergeometric test following FDR. Values > = 0.05 are considered significant. The full names of the metabolites are listed at Table 1. Additional file 3:
**Is a figure showing metabolites versus genes correlation matrix (Spearman’s rho coefficient).** The heatmap displays correlation values for all 77 metabolic traits detailed at Figure 2. Out of 27,427 unigenes, 9721 genes were significantly correlated with at least a single metabolic trait (|*r*| > 0.3). The rows in the heat map are the genes clustered by their expression patterns and the columns are the metabolic traits. Additional file 4:
**Is a table listing results from one-way ANOVA and Broad-sense heritability (H** ^**2**^ **) analyses for the 77 metabolic traits.** Asterisks indicate a square root transformation of the raw data. Additional file 5:
**Is a table listing the level of metabolites’ accumulation across RILs.** The full names of the metabolites are listed in Table 1. Additional file 6:
**Is a table listing the level of genes’ expression (RPKM) across RILs.**
