# Supplementary material for: Systems approach for exploring the intricate associations between sweetness, color and aroma in melon fruits
Source: BMC Plant Biol. 2015 Mar 3;15:71. doi: 10.1186/s12870-015-0449-x (PMC4448286; doi:10.1186/s12870-015-0449-x)
Supplement: Additional file 1: — Provides a comparison of the intra- and inter-cluster similarities of metabolite accumulation patterns in the three clusters of metabolites, determined according to hierarchical clustering. [file 12870_2015_449_MOESM1_ESM.docx]

**Comparison of intra- and inter-cluster values in the metabolites versus metabolites correlation matrix**

A metabolite versus metabolite correlation matrix was constructed, and three key clusters were predicted as described in the main text and visualized in Figure 2B. To evaluate the integrity of the clusters, we compared the distribution of correlation values (providing an estimate for the similarity in the pattern of accumulation across the RIL population), within and between clusters.

Differences in the intra- and inter-cluster correlation values are shown in Table 1, indicating that metabolites within the same cluster show significantly higher similarity in their pattern of accumulation.

Significant associations in the metabolite versus metabolite matrix were filtered according to a cutoff of |r| > 0.3 (main text). Positive associations are frequent between metabolites that are classified to the same cluster; negative associations were detected only between members of different clusters (Table 2). Notably, a higher number of associations is detected between metabolites from the closely connected Clusters II and III, in comparisons to the lower number of associations formed between these cluster members and metabolites from Cluster I. Similarly, all significant negative associations are detected between members of Cluster I and members of cluster II and III.

|  | Cluster I | Cluster II | Cluster III |
| --- | --- | --- | --- |
| Cluster I | 0.33 | -0.14 [1.2e-120] | 0.04 [2.6e-113] |
| Cluster II | -0.14 [4e-66] | 0.28 | 0.04[1.2e-31] |
| Cluster III | 0.04 [1.7e-102] | 0.04[2.1e-71] | 0.19 |

Table 1. Distribution of intra- and inter-cluster correlation values. Values in red and green indicate the mean intra- and inter-cluster correlation. Numbers in brackets indicate the significance of the difference in the distribution of the correlation values between the intra-cluster correlation values (row cluster) and inert-cluster correlation values (column cluster). Significance was determined according to the *P* value in a wilcoxon two-sided test.

|  | Cluster I | Cluster II | Cluster III |
| --- | --- | --- | --- |
| Cluster I | 93 | 8 / 61 | 15 / 26 |
| Cluster II |  | 89 | 47 |
| Cluster III |  |  | 151 |

Table 2. Number of significant positive and negative associations between cluster members and between members of different clusters. Associations were set according to threshold of correlation coefficient rho >= |0.3| (*p* value < 0.003). Numbers in red and green indicate positive and negative correlations, respectively.
