# Supplementary figures and images for: Systems approach for exploring the intricate associations between sweetness, color and aroma in melon fruits
Source: BMC Plant Biol. 2015 Mar 3;15:71. doi: 10.1186/s12870-015-0449-x (PMC4448286; doi:10.1186/s12870-015-0449-x)

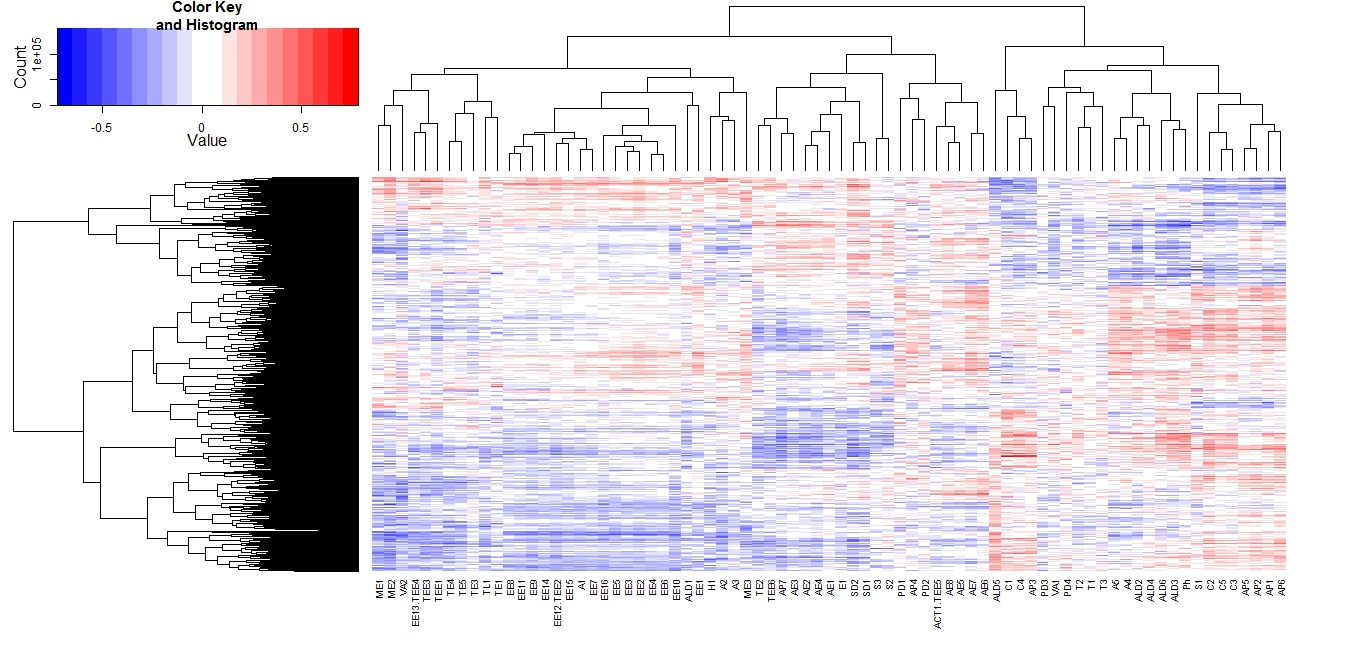

Supplement: Additional file 3: — Is a figure showing metabolites versus genes correlation matrix (Spearman’s rho coefficient). The heatmap displays correlation values for all 77 metabolic traits detailed at Figure 2. Out of 27,427 unigenes, 9721 genes were significantly correlated with at least a single metabolic trait (|r| > 0.3). The rows in the heat map are the genes clustered by their expression patterns and the columns are the metabolic traits. [file 12870_2015_449_MOESM3_ESM.jpeg]
